# Enhanced Lymphoma Subtype Classification and Prognosis Using Machine Learning with ^18^F-FDG PET/CT Radiomics: Beyond SUV_max_

**DOI:** 10.1007/s13139-026-01017-4

**Published:** 2026-04-27

**Authors:** Setareh Hasanabadi, Seyed Mahmud Reza Aghamiri, Ahmad Ali Abin, Habibeh Vosoughi, Farshad Emami, Mehrdad Bakhshayesh Karam, Marzieh Nejabat, Abtin Dorudinia, Hossein Arabi, Habib Zaidi

**Affiliations:** 1 Department of Medical Radiation Engineering, Shahid Beheshti University, Tehran, Iran; 2 Faculty of Computer Science and Engineering, Shahid Beheshti University, Tehran, Iran; 3 Research Center for Nuclear Medicine, Tehran University of Medical Sciences, Tehran, Iran; 4 Razavi Cancer Research Center, Razavi Hospital, Imam Reza International University, Mashhad, Iran; 5 Radiology Department, Shahid Beheshti University of Medical Sciences, Tehran, Iran; 6 Shahid Beheshti University of Medical Sciences, National Research Institute of Tuberculosis and Lung Diseases, Tehran, Iran; 7 Nuclear Medicine Department, Masih Daneshvari Hospital, Tehran, Iran; 8 Department of Radiology and Nuclear Medicine, Medical University of Vienna, Vienna, Austria; 9 Keck School of Medicine, University of Southern California, Los Angeles, CA USA; 10 Division of Nuclear Medicine & Molecular Imaging, Geneva University Hospital, Geneva, CH-1211 Switzerland; 11 Department of Nuclear Medicine and Molecular Imaging, University of Groningen, University Medical Center Groningen, Groningen, 9700 RB Netherlands; 12 Department of Nuclear Medicine, University of Southern Denmark, Odense, 500 Denmark; 13 University Research and Innovation Center, Óbuda University, Budapest, Hungary

**Keywords:** Lymphoma, ^18^F-FDG PET/CT, Radiomics, Classification, Machine learning, Extra-nodal differentiation, Survival analysis

## Abstract

**Background:**

This study explored a machine learning approach using ^18^F-FDG PET/CT as a non-invasive alternative to biopsy, incorporating tumor-to-liver ratio (TLR) PET radiomics, and performed survival analysis to improve lymphoma management.

**Methods:**

In this cohort study, baseline ^18^F-FDG PET/CT scans of newly diagnosed, histologically confirmed lymphoma patients were analyzed. Lesions were segmented using 3D Slicer, and radiomic features were extracted and normalized by tumor-to-liver ratios. Patient-level features were used to train three machine learning models (XGBoost, AdaBoost, Logistic Regression) using nested cross-validation with SMOTE for class balancing. A model based on SUV_max_ metrics served as baseline. Radiomic features were also evaluated for correlation with 3- and 5-year survival using the Mann-Whitney U test.

**Results:**

A total of 156 lymphoma patients were analyzed, with 2,076 lesions segmented and 200 radiomic features extracted. For subtype classification, AdaBoost achieved the highest AUC for Diffuse Large B-cell (DLBCL) (0.863, accuracy 0.742), while XGBoost performed best for High-Grade Non-Hodgkin lymphoma (NHL) (AUC 0.825, accuracy 0.735) and Nodular Sclerosis Hodgkin Lymphoma (NS-HL) (AUC 0.827, accuracy 0.832). Logistic regression showed the best results for Classical Hodgkin Lymphoma (C-HL) (AUC 0.849, accuracy 0.775). The SUV_max_-based model (LR-SUV_MAX) consistently underperformed (AUCs: C-HL 0.630, High-Grade NHL 0.700, NS-HL 0.638, DLBCL 0.664), with all differences being statistically significant (*p* < 0.001). Radiomic and clinical features including SUV-GLSZM small area emphasis (*p* = 0.0019), age (*p* = 0.0002), and spleen involvement (*p* = 0.0014) were significantly associated with 3- and 5-year overall survival in 110 and 74 patients, respectively.

**Conclusion:**

Radiomic features combined with machine learning significantly improve lymphoma subtype classification over SUV_max_ alone and show potential for predicting patient survival.

**Supplementary Information:**

The online version contains supplementary material available at https://doi.org/10.1007/s13139-026-01017-4.

## Introduction

Lymphoma is the most common hematological malignancies in the world accounting up to 5% of all cancer cases [1, 2]. Lymphomas can be generically categorized as 10% Hodgkin lymphoma (HL) and 90% Non-Hodgkin lymphoma (NHL) [3]. In addition, HL is divided into classical and non-classical varieties, whereas NHL is divided into types of B-, T-, and natural killer (NK) cells [4].

For the diagnosis of the majority of hematopoietic and lymphoid tissue malignancies, pathological examination of the affected tissue—typically obtained through surgical resection—remains the gold standard [5]. The limitations of this approach include obtaining an insufficient amount of tissue for accurate diagnosis, subjective interpretation, procedural complications, such as bleeding, damage to nearby organs, etc., and spatial limitation to a single tissue, make it important to think about alternative methods. Today’s improvement in personalized therapy is leading to longer survival and increasing the possibility of recurrence, which again must be proven by biopsy [6].

As a non-invasive method, positron emission tomography (PET) in conjunction with computed tomography using ^18^F-fluorodeoxyglucose (^18^F-FDG PET/CT) is widely utilized for the initial assessment and re-staging of lymphoma patients, as well as for post-treatment evaluation, therapy monitoring, and follow-up, providing three-dimensional visualization of metabolic abnormalities. Despite its well-established benefits and potentials, it also bears limitations, such as false positives and false negatives [7–10].

The maximum standardized uptake value (SUV_max_), as a quantitative parameter reflecting the metabolic state of cancer cells, is the most widely used PET parameter, especially in the monitoring of treatment response, but it has been shown that different lymphoma subtypes have variability in FDG avidity [11, 12]. Single-voxel representation and sensitivity to artefactual noise can also affect SUV_max_ [13, 14]. Recent radiomic techniques leverage extensive quantitative features from medical images, potentially providing a more accurate representation of in vivo tumor conditions compared to SUV_max_ alone [15–18]. A number of studies showed that ^18^F-FDG PET/CT radiomics can effectively differentiate lymphoma from other cancer types [19–23] and other lymphoma subtypes, enhancing their effectiveness over SUV_max_ alone [24–27]. Radiomics analysis is commonly combined with machine learning techniques for classification tasks [28–31].

To the best of our knowledge, there is a lack of studies exploring the potential of combining ^18^F-FDG PET/CT Tumor-to-Liver Ratio (TLR) radiomics with CT and clinical data across different subtypes of lymphoma.

This study investigated the potential of utilizing multimodal radiomics and machine learning to analyze TLR PET values along with CT features extracted from baseline ^18^F-FDG PET/CT scans, combined with selected baseline clinical data, to assist in the classification of lymphoma subtypes. This approach aims to provide a non-invasive tool that may support clinical decision-making and potentially reduce the need for repeated biopsies in certain cases, offering insights for personalized treatment. Additionally, survival analysis was performed to identify radiomic features associated with overall patient survival at three- and five-year time points.

## Materials and Methods

### Study Design

This is a retrospective cohort study conducted at Masih Daneshvari Hospital from 2014 to 2024. The research was approved by the Medical Ethical Review Committee of Shahid Beheshti University of Medical Sciences under the ethical code IR.SBMU.NRITLD.REC.1402.060. Informed consent from all participants was waived by the Medical Ethics Review Committee due to the non-interventional design of the study. Patients with newly diagnosed lymphoma who underwent a baseline ^18^F-FDG PET/CT scan and had histopathological confirmation of their initial diagnosis were included in this study.

### Patient Selection Criteria

Patients with negative/suspect results, non-original cases, suspected concurrent infection, known fibrotic liver disease that interferes with normal liver uptake, such as cirrhosis, concurrent malignancy or any recent malignancy, such as breast cancer, were excluded from the study. Furthermore, PET/CT images of patients affected by motion artefacts, artefacts in pathological areas were also excluded. Additional details, such as height, weight, and injected dose, were extracted from the DICOM files, while age, sex, biopsy diagnosis, and biopsy site information were obtained from pathology reports.

### Histopathologic Reports: Sample Collection and Analysis

All types of tissue biopsies (fine needle aspiration, core biopsy, incisional and excisional biopsies) from all organs were included. Routine tissue procedures with H&E staining and immunostaining for CD-10 and possibly other CDs (CD3, CD 20, CD 19, … etc.) to differentiate lymphoma subtypes were performed according to the same protocol, but interpreted by different pathologists in the hospital. Classification was performed according to the recent WHO classification of hematopoietic and lymphoid tissues [5].

### ^18^F-FDG Production Protocol

^18^F-FDG production commences with the utilization of the GE Healthcare MINItrace Qilin positron-emitting isotope production system, operating at 9.6 MeV energy, with a module efficiency ranging from 45% to 55%. The target (^18^F-^25^Nb) undergoes bombardment for typically one and a half to two hours. Operational guidelines for FDG production were adhered to using the TRACERlab MX_FDG.

### PET/CT Imaging Protocol

PET/CT scans were performed on Discovery 690 GE scanners, equipped with a 64-slice CT and Time-of-Flight (TOF) capability. Whole-body PET/CT scans were performed from the vertex to mid-thigh. PET images were reconstructed using the Q.Clear algorithm, referred to as VUE Point HD/FX reconstruction. The injected ^18^F-FDG activity ranged from 99.46 to 520.68 MBq (approximately 3×weight), averaging at 331.148 MBq, calculated by subtracting the remaining dose in the syringe from the calibrated dose. The average uptake time was 60 min with a range of 45 to 75 min. The duration of each PET imaging bed position was two to three minutes. The PET scan slice thickness was 3.27 mm, but the low dose CT slice thickness varied from 1.33 to 2.5 mm. The X-ray tube current was adjusted automatically by the Smart mAs algorithm according to patient weight (50 to 150 mA). The tube voltage was set to 120 kVp with the helical pitch factor maintained at 0.9. ^18^F-FDG PET images were corrected for scatter and attenuation using CT data.

### Primary Image Evaluation and Lesion Segmentation

Initially, PET/CT images underwent evaluation by a nuclear medicine physician with over 10 years of experience. The assessment included evaluating stage, nodal, and extra nodal involvement. In cases where extra nodal involvement was identified, the physician also assessed which organ/s was/were involved. Subsequently, lesions were delineated by a nuclear medicine physician using a semi-automated graphical-based method [32], integrated as an extension for the 3D Slicer software [33]. The physician then made any necessary adjustments to the lesion borderlines. To enhance comparability across patients and mitigate scanner- and patient-specific SUV variability, we normalized all lesion metrics using the tumor-to-liver ratio (TLR). Unlike absolute SUV-based normalization, TLR incorporates an internal physiological reference (healthy liver tissue), which is metabolically stable and minimally influenced by inter-scan variability. This approach has been shown to increase feature robustness and reduce dependence on absolute SUV calibration.

**Fig. 1:**
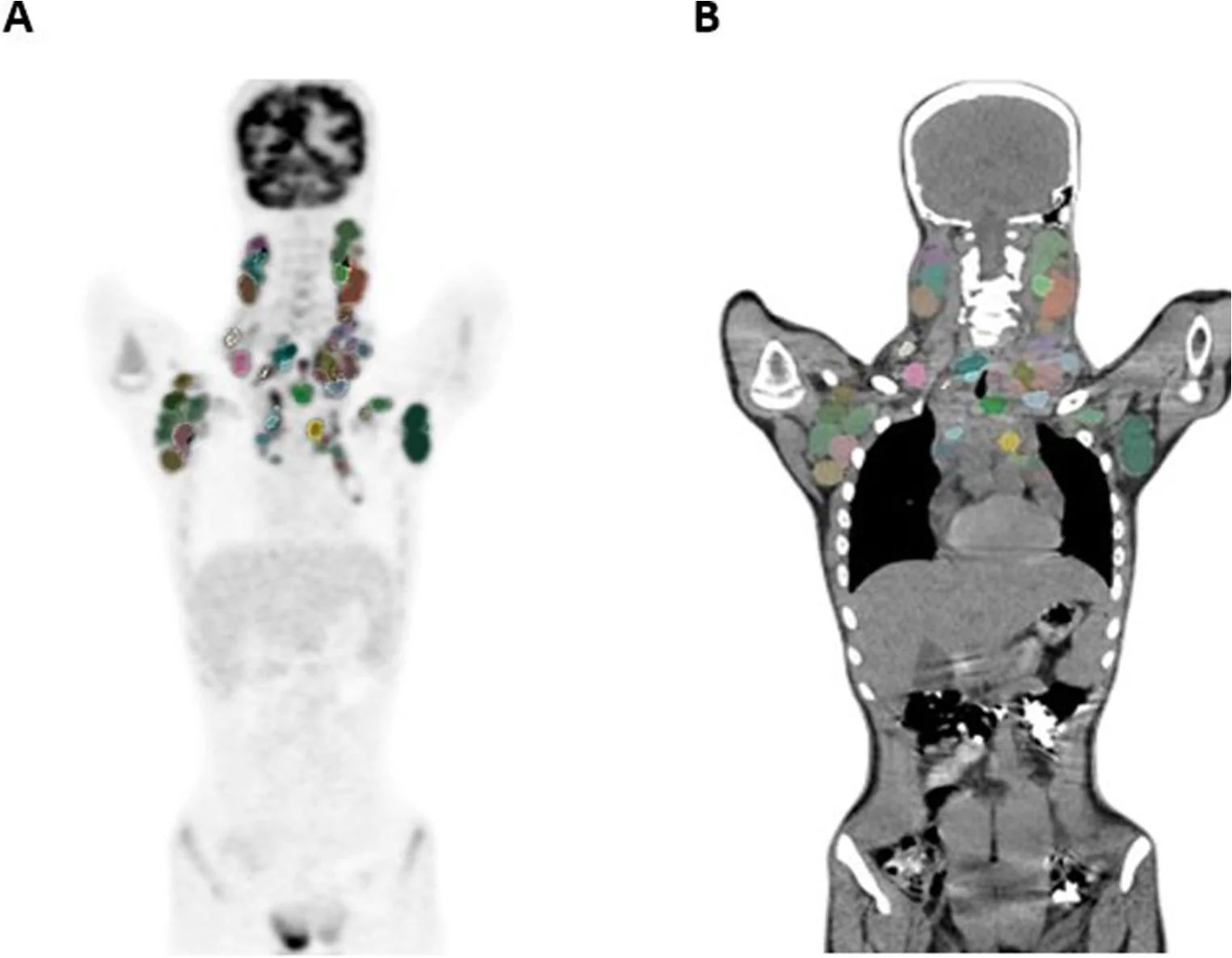
Representative example of the semi-automatic graph-based segmentation method for a patient with stage 2 Hodgkin lymphoma nodular sclerosis type in the coronal view, with the sample taken from the patient’s cervical region for pathological examination. (**A**) PET image, (**B**) CT image

Importantly, we would like to stress that, aside from lesions listed in the criteria, we included all tumors in the body of each patient, without any numerical limitations. Following the delineation of the lesions, a 3-dimensional region of interest (ROI) was defined on the right lobe of the liver, where no lesions were present, in accordance with the PERCIST criteria [34], for calculating TLR radiomics. A representative example of the outcome of image segmentation is illustrated in Fig. 1.

### Image Preprocessing and Features Extraction

**Fig. 2:**
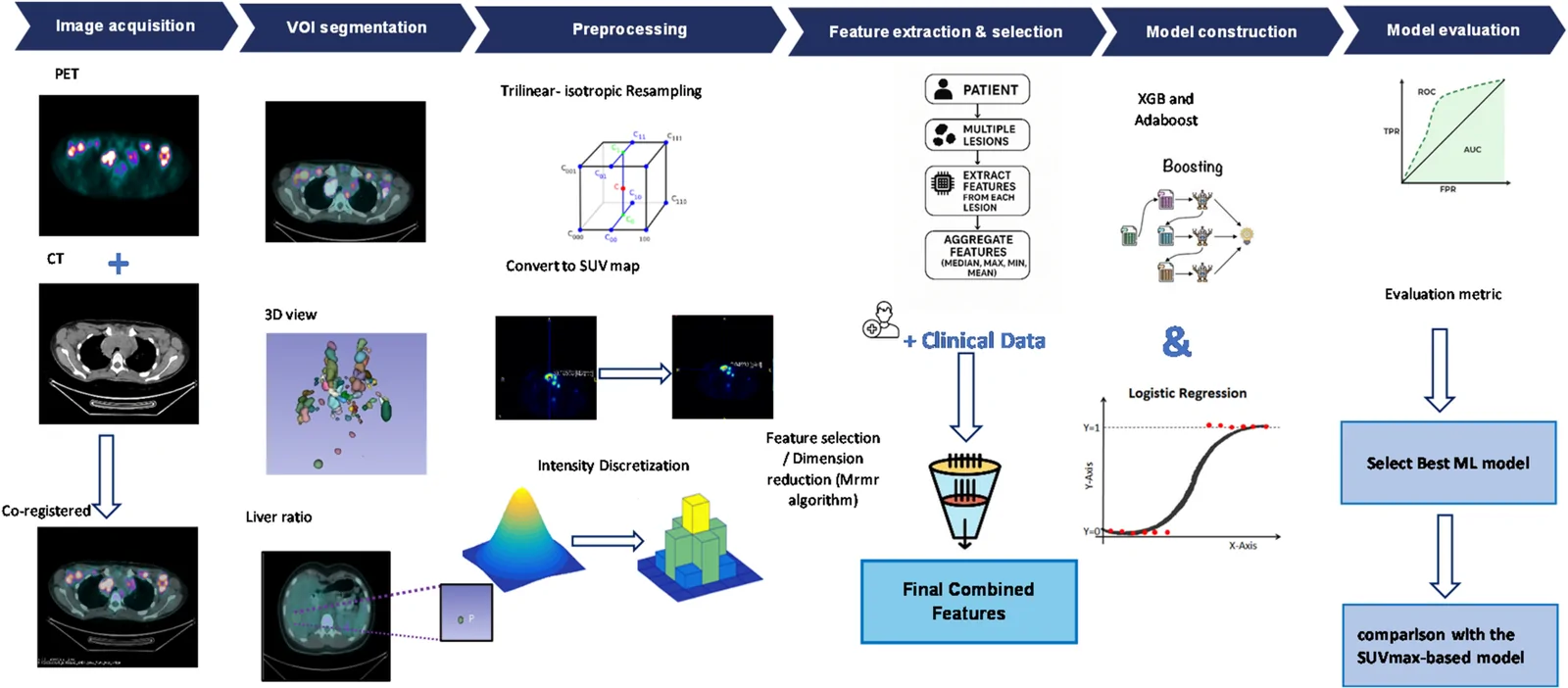
Radiomics workflow. ^18^F-FDG PET and CT images were first acquired and then preprocessed and segmented. Radiomic features were extracted from the segmented lesions. Machine learning classifiers were trained using radiomic features from ^18^F-FDG PET and CT, along with shape features and clinical data. The model with the highest AUC was selected as the best combined model and compared to the SUV_max_-based model

As shown in Fig. 2, the process begins with resampling images to ensure isotropic voxel spacing using trilinear interpolation, maintaining feature consistency across rotations and different scanners. Low-dose CT images were resampled to 1 × 1 × 1 mm³ and PET images to 2 × 2 × 2 mm³ voxel size. Subsequently, PET images were converted to SUV maps. SUV maps and CT images were discretized using a fixed bin size of 0.25 SUV and five Hounsfield units according to the guidelines set by the Image Biomarker Standardization Initiative (IBSI) [35], respectively, and then, using the PyRadiomics extension [36] within the 3D Slicer software [33]. Radiomic features were extracted separately from each tumor in a patient, and then aggregated using four statistical measures (minimum, maximum, mean, and median) to create a single feature representation per patient. Clinical features (e.g., age, gender, disease stage, and extranodal involvement in organs such as bone, soft tissue, spleen, bone marrow, stomach, liver, lung, adrenal gland, colon, and parietal regions) were added to the radiomic feature set before feature selection.

To calculate the TLR of feature values, we extracted features from a 3-dimensional region of interest (ROI) defined on the right lobe of the liver, where no lesions were present, using PET images and excluding shape features. This normalization against healthy liver tissue helps reduce patient variability and improve the comparability of tumor features. TLR normalization was applied at the feature level only, after extraction of all radiomic features (including high-order texture features, such as GLCM, GLSZM, GLRLM, GLDM, and NGTDM) from the discretized SUV maps and before patient-level aggregation using minimum, maximum, mean, and median values.

Since some patients had multiple tumors and our analysis was conducted at the patient level, radiomic features were first extracted separately for each lesion. To account for inter-patient variability, we normalized each lesion feature using the tumor-to-liver ratio (TLR), calculated by dividing the lesion feature (e.g., SUV_max_) by the corresponding value from a lesion-free spherical region of the liver (3 cm diameter). Following TLR normalization, lesion-level features were combined into patient-level metrics using the mean, maximum, minimum, and median, capturing both typical and extreme characteristics of each patient’s lesions. This strategy provides a comprehensive and reproducible representation of patients with multiple lesions.

### Machine Learning Elaboration

A nested cross-validation framework was employed to robustly evaluate model performance and select relevant features. The outer loop utilized a 5-fold stratified cross-validation (StratifiedKFold, *n* = 5, random_state = 42) to split the data into training and test sets while preserving class balance. Within each outer fold, a 3-fold stratified cross-validation (*n* = 3, random_state = 42) was performed for hyperparameter tuning and feature selection. This nested design helps reduce overfitting and provides an unbiased estimate of model generalization.

Feature selection was conducted within each inner fold by identifying the most informative features tailored to each model: for Logistic Regression, absolute values of the coefficients were used, while for tree-based models (XGBoost and AdaBoost), built-in feature importance scores were applied. Only features with normalized importance exceeding the 75th percentile were retained. To address class imbalance, the Synthetic Minority Oversampling Technique (SMOTE) with a fixed random seed (42) was applied within each training fold to generate synthetic samples of the minority class, ensuring balanced datasets for model training.

Three machine learning models—XGBoost, Logistic Regression, and AdaBoost—were evaluated. Hyperparameters were optimized using grid search within the inner cross-validation loop, with the area under the ROC curve (AUC) as the scoring metric. The hyperparameter grids were as follows: for XGBoost, number of estimators (50 or 100), maximum tree depth (3 or 5), and learning rate (0.01 or 0.1); for Logistic Regression, regularization strength (C values of 0.1, 1.0, or 10.0) and penalty type (L1 or L2); and for AdaBoost, number of estimators (50 or 100) and learning rate (0.01 or 0.1). Additionally, due to SUV_max_’s widespread use in ^18^F-FDG PET/CT studies, a Logistic Regression model (LogisticRegression_SUV_MAX) was trained without feature selection, using a predefined set of four SUV-based features (minimum, mean, maximum, and median of SUV_MAX), serving as a baseline comparison.

Model performance was assessed using multiple metrics, including accuracy, F1-score, sensitivity, specificity, and AUC. Predictions and probability scores were generated on the test sets for each outer fold. To quantify uncertainty, 95% confidence intervals for each metric were estimated using bootstrapping with 1,000 iterations, which involved resampling test set predictions with replacement to build metric distributions. To focus on the most reliable features, we included only those appearing in at least two of the five outer folds of the nested cross-validation. The mean and standard deviation of feature importance values were then calculated across these folds to summarize their stability and relevance.

For statistical comparison of models, the DeLong test was used to compare AUC values pairwise, assessing whether differences in discriminative ability were significant (*p* < 0.05). The test calculates a Z-statistic and corresponding p-value based on the variance and covariance of predicted probabilities.

All analyses were performed using Python version 3.11.13. Core libraries included scikit-learn 1.6.1 for machine learning and evaluation, numpy 2.0.2 for numerical operations, pandas 2.2.2 for data handling, xgboost for the XGBoost model, imblearn 0.13.0 for SMOTE, scipy for statistical testing, and matplotlib 3.10.0 alongside seaborn for data visualization.

### Survival Analysis

Given that our dataset allowed us to perform survival analysis of the patients, we designed an additional experiment for this purpose. Patients were divided into two groups: those who survived and those who did not, in order to achieve the goal of identifying radiomic features that demonstrate significant correlations with patient survival over these predetermined time intervals. The survival time of survivors was computed from the pathological diagnosis date to the last follow-up date, whereas the survival time of non-survivors was calculated from the pathological diagnosis date to the date of death. Using the Mann-Whitney U test and statistical analysis, we investigated the relationship between radiomic features and patient survival at three and five years. Due to the relatively low number of events in this cohort, survival analyses were kept exploratory using Mann-Whitney U tests at fixed time points. Future larger studies should apply proper time-to-event techniques, such as Kaplan-Meier curves or Cox regression, to better account for censoring and follow-up duration. The statistical significance of the associations was established at a significance level of 0.05. SciPy 1.11.4 and Python 3.10.12 were used in the analysis.

## Results

### Patient Demographics

This study examined 156 patients with various types of lymphoma: 51 with Diffuse Large B-cell lymphoma (DLBCL), 78 with C-HL, 49 with NS-HL, 55 with HG-NHL, and 23 with rare lymphoma types grouped under the “Others” category. Within the “Others” subgroup, there were 9 cases of Nodular lymphocyte predominant, 1 case of mantle cell lymphoma, 4 cases of follicular lymphoma, 7 cases of marginal zone, 1 case of cutaneous t-cell, 1 case of low-grade B cell, and 4 cases of nodular predominant HL. Table 1 provides additional information about patients’ characteristics.

**Table 1:** Patient demographics within the first biopsy site (*n* = 156)

	DLBCL	C-HL	NS-HL	HG-NHL	Others
Gender					
Male	33	44	23	37	12
Female	18	34	26	18	11
Age (years)					
Median	54	28	29	52	49
Ann arbor staging					
I	9	4	0	9	4
II	16	45	29	17	9
III	8	12	7	8	6
IV	18	17	13	21	4
Extra nodal involvement					
Yes	23	23	18	26	8
No	28	55	31	29	15
Extra nodal site					
Bone	12	10	7	15	2
Soft tissue	10	4	4	10	2
Spleen	9	13	10	10	4
Bone marrow	4	7	7	4	-
Stomach	1	-	-	1	-
Liver	4	5	5	5	-
Lung	2	5	3	2	1
adrenal	-	1	1	-	-
Colon	1	-	-	1	-
subcutaneous	1	-	-	1	1
parietal	1	-	-	1	-
Total	51	78	49	55	23

Abbreviations: C-HL: Classical Hodgkin Lymphoma, NS-HL: Nodular Sclerosis Hodgkin Lymphoma, HG-NHL: High-Grade Non-Hodgkin Lymphoma, DLBCL: Diffuse Large B-Cell Lymphoma. The cohort consisted of 156 unique patients. The sum of diagnostic category counts in Table 1 exceeds this number because certain cases met criteria for more than one diagnostic classification (e.g., overlap between DLBCL and HG-NHL). Therefore, category frequencies represent diagnostic occurrences rather than mutually exclusive patient counts. The table caption has been updated to reflect this clarification

### Data Visualization/Interpretation

**Fig. 3:**
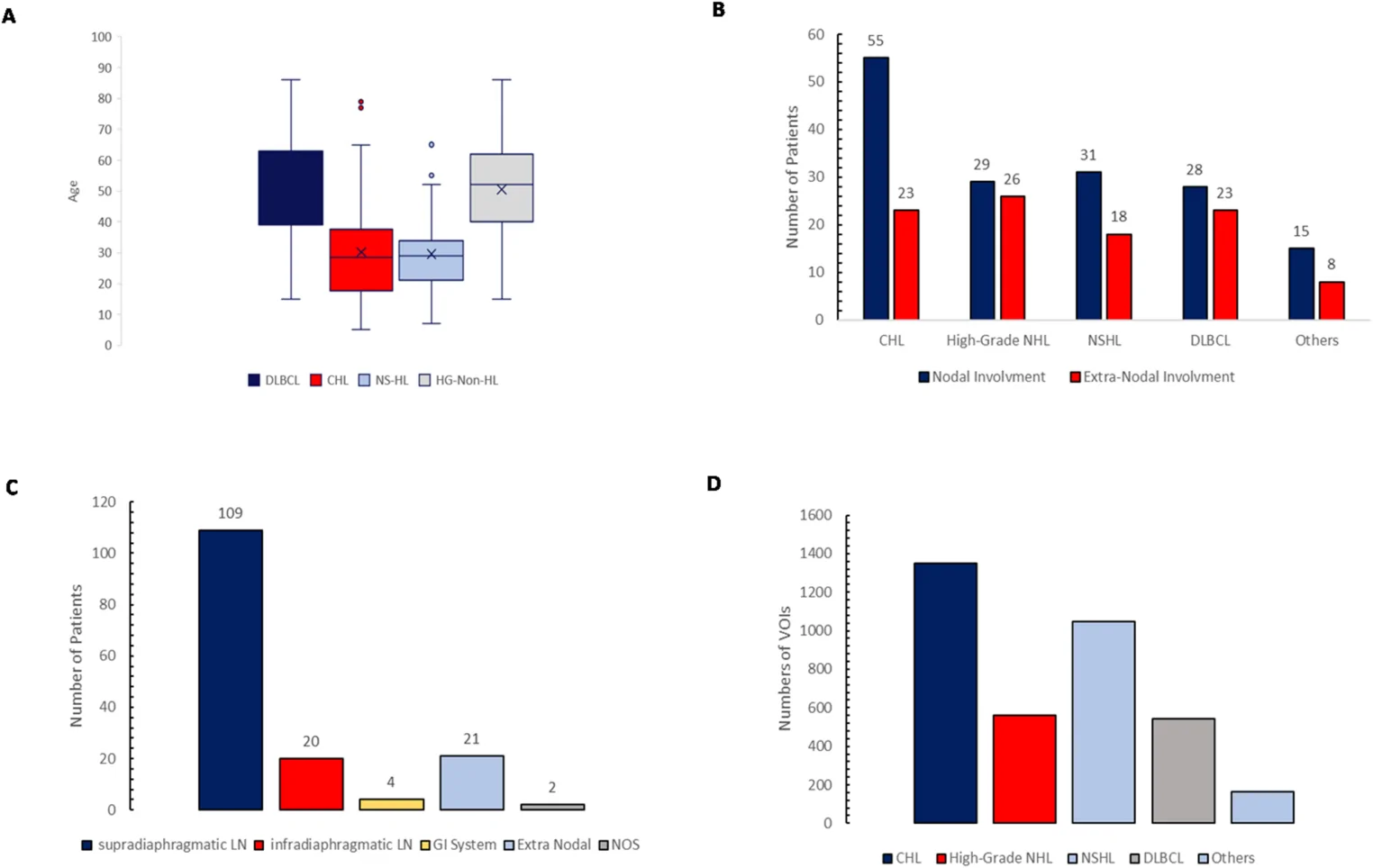
(**A**) Age distribution by pathology diagnosis, (**B**) The number of patients by pathology diagnosis with nodal versus extranodal involvement, (**C**) Total segmented VOI in each subtype, (**D**) Number of patients by biopsy region

The ANOVA test revealed a significant difference in patient ages across pathology diagnoses (p-value < 0.0001). As shown in Fig. 3A, patients in the C-HL group were notably younger compared to other subtypes. Figure 3B demonstrates the distribution of nodal versus extranodal involvement by pathology diagnosis: HL cases primarily presented with nodal involvement, whereas high-grade non-Hodgkin lymphomas (e.g., DLBCL) showed a higher prevalence of extranodal disease. However, no statistically significant differences were observed in gender distribution or disease stage across groups (p-values = 0.45 and 0.65, respectively). Finally, Fig. 3C illustrates that sampling sites were predominantly located in the cervical and inguinal regions.

### Subtype Differentiation

As shown in Fig. 3D, from a total of 2,076 segmented lesions—including 543 DLBCL, 1,049 NS-HL, 1,351 C-HL, 561 HG-NHL, and 164 others—a total of 200 radiomic features were extracted. These features included 14 shape features and 93 features from each imaging modality.

Nested cross-validation was performed to evaluate the performance of four classification models—XGBoost, Logistic Regression (LR), AdaBoost, and Logistic Regression with SUV_max_ features (LR-SUV_MAX)—in discriminating lymphoma subtypes. The models were trained and tested on four lymphoma classification tasks: C-HL), High-Grade NHL, (NS-HL, and DLBCL versus other lymphoma types.

For C-HL classification (Table 2), Logistic Regression and AdaBoost demonstrated superior performance with mean accuracies of 0.775 ± 0.042 and 0.769 ± 0.048, respectively, while XGBoost closely followed at 0.756 ± 0.032. The F1-scores and AUCs were consistent, with LR and AdaBoost achieving approximately 0.77 and 0.85, respectively. Sensitivity peaked for AdaBoost (0.818 ± 0.116), and specificity was comparable among XGBoost and LR (~ 0.78). The LR-SUV_MAX model, relying solely on SUV_max_ features, showed markedly poorer results with an accuracy of 0.638 ± 0.025 and low specificity (0.493 ± 0.011), despite moderate sensitivity (0.782 ± 0.052). Pairwise DeLong’s tests indicated statistically significant performance improvements for XGBoost, LR, and AdaBoost compared to LR-SUV_MAX (*p* < 0.001), while differences among the former three were not significant.

**Table 2:** Nested cross-validation performance metrics for lymphoma subtype classification

Subtype	Model	Metric	Mean ± SD	95% CI	# Selected Features
C-HL	XGBoost	Accuracy	0.756 ± 0.032	[0.686–0.821]	131.0 ± 13.9
		F1	0.748 ± 0.045	[0.667–0.821]	
		AUC	0.846 ± 0.046	[0.775–0.897]	
		Sensitivity	0.729 ± 0.080	[0.625–0.828]	
		Specificity	0.783 ± 0.085	[0.684–0.870]	
	LogisticRegression	Accuracy	0.775 ± 0.042	[0.699–0.840]	12.4 ± 1.9
		F1	0.770 ± 0.058	[0.684–0.844]	
		AUC	0.849 ± 0.066	[0.782–0.913]	
		Sensitivity	0.767 ± 0.103	[0.663–0.859]	
		Specificity	0.782 ± 0.052	[0.682–0.870]	
	AdaBoost	Accuracy	0.769 ± 0.048	[0.699–0.833]	18.4 ± 2.2
		F1	0.776 ± 0.058	[0.704–0.846]	
		AUC	0.840 ± 0.058	[0.773–0.901]	
		Sensitivity	0.818 ± 0.116	[0.725–0.896]	
		Specificity	0.718 ± 0.105	[0.620–0.815]	
	LogisticRegression_SUV_MAX	Accuracy	0.638 ± 0.025	[0.532–0.686]	All
		F1	0.681 ± 0.033	[0.581–0.740]	
		AUC	0.630 ± 0.011	[0.514–0.705]	
		Sensitivity	0.782 ± 0.052	[0.671–0.863]	
		Specificity	0.493 ± 0.011	[0.342–0.565]	
High-Grade NHL	XGBoost	Accuracy	0.735 ± 0.075	[0.665–0.806]	138.2 ± 17.6
		F1	0.624 ± 0.116	[0.518–0.730]	
		AUC	0.825 ± 0.066	[0.746–0.877]	
		Sensitivity	0.636 ± 0.163	[0.500–0.755]	
		Specificity	0.790 ± 0.058	[0.701–0.872]	
	LogisticRegression	Accuracy	0.690 ± 0.097	[0.619–0.761]	173.2 ± 68.9
		F1	0.569 ± 0.133	[0.458–0.677]	
		AUC	0.775 ± 0.099	[0.690–0.843]	
		Sensitivity	0.582 ± 0.159	[0.455–0.712]	
		Specificity	0.750 ± 0.089	[0.667–0.837]	
	AdaBoost	Accuracy	0.723 ± 0.101	[0.652–0.794]	26.4 ± 2.6
		F1	0.617 ± 0.136	[0.505–0.723]	
		AUC	0.835 ± 0.071	[0.771–0.897]	
		Sensitivity	0.636 ± 0.163	[0.509–0.750]	
		Specificity	0.770 ± 0.093	[0.687–0.857]	
	LogisticRegression_SUV_MAX	Accuracy	0.708 ± 0.029	[0.665–0.800]	All
		F1	0.563 ± 0.039	[0.483–0.704]	
		AUC	0.700 ± 0.017	[0.628–0.810]	
		Sensitivity	0.543 ± 0.046	[0.431–0.694]	
		Specificity	0.797 ± 0.036	[0.752–0.902]	
NS-HL	XGBoost	Accuracy	0.832 ± 0.038	[0.774–0.884]	134.2 ± 12.1
		F1	0.731 ± 0.029	[0.628–0.821]	
		AUC	0.827 ± 0.056	[0.736–0.897]	
		Sensitivity	0.713 ± 0.100	[0.595–0.833]	
		Specificity	0.887 ± 0.098	[0.822–0.943]	
	LogisticRegression	Accuracy	0.677 ± 0.035	[0.613–0.755]	100.2 ± 61.3
		F1	0.536 ± 0.051	[0.423–0.645]	
		AUC	0.747 ± 0.046	[0.646–0.804]	
		Sensitivity	0.593 ± 0.083	[0.460–0.730]	
		Specificity	0.718 ± 0.055	[0.629–0.800]	
	AdaBoost	Accuracy	0.787 ± 0.033	[0.723–0.852]	20.6 ± 1.9
		F1	0.690 ± 0.043	[0.584–0.788]	
		AUC	0.831 ± 0.058	[0.752–0.900]	
		Sensitivity	0.753 ± 0.107	[0.630–0.872]	
		Specificity	0.801 ± 0.077	[0.726–0.877]	
	LogisticRegression_SUV_MAX	Accuracy	0.592 ± 0.051	[0.561–0.710]	All
		F1	0.501 ± 0.038	[0.396–0.623]	
		AUC	0.638 ± 0.059	[0.563–0.751]	
		Sensitivity	0.639 ± 0.019	[0.478–0.750]	
		Specificity	0.572 ± 0.071	[0.557–0.743]	
DLBCL	XGBoost	Accuracy	0.710 ± 0.074	[0.639–0.781]	136.2 ± 27.6
		F1	0.593 ± 0.061	[0.465–0.692]	
		AUC	0.803 ± 0.086	[0.742–0.877]	
		Sensitivity	0.629 ± 0.061	[0.489–0.750]	
		Specificity	0.749 ± 0.103	[0.664–0.829]	
	LogisticRegression	Accuracy	0.716 ± 0.131	[0.645–0.787]	122.8 ± 73.4
		F1	0.605 ± 0.104	[0.460–0.689]	
		AUC	0.722 ± 0.129	[0.637–0.807]	
		Sensitivity	0.609 ± 0.050	[0.471–0.732]	
		Specificity	0.766 ± 0.190	[0.692–0.846]	
	AdaBoost	Accuracy	0.742 ± 0.058	[0.671–0.806]	26.6 ± 3.6
		F1	0.633 ± 0.089	[0.527–0.731]	
		AUC	0.863 ± 0.057	[0.784–0.906]	
		Sensitivity	0.689 ± 0.151	[0.556–0.808]	
		Specificity	0.769 ± 0.059	[0.689–0.848]	
	LogisticRegression_SUV_MAX	Accuracy	0.708 ± 0.041	[0.658–0.794]	All
		F1	0.540 ± 0.050	[0.449–0.679]	
		AUC	0.664 ± 0.065	[0.586–0.779]	
		Sensitivity	0.535 ± 0.053	[0.409–0.686]	
		Specificity	0.793 ± 0.041	[0.740–0.885]	

Description: Mean ± standard deviation (SD) and 95% confidence intervals (CI) for accuracy, F1-score, area under the receiver operating characteristic curve (AUC), sensitivity, and specificity across five outer folds for XGBoost, Logistic Regression (LR), AdaBoost, and Logistic Regression with SUV features (LR-SUV_MAX) in classifying Classical Hodgkin Lymphoma (C-HL), High-Grade Non-Hodgkin Lymphoma (High-Grade NHL), Nodular Sclerosis Hodgkin Lymphoma (NS-HL), and Diffuse Large B-Cell Lymphoma (DLBCL) versus other lymphomas. The mean ± SD of selected features is reported; "All" indicates no feature selection for LR-SUV_MAX. Metrics are derived from nested cross-validation, with CIs calculated via bootstrap on the full test set

**Fig. 4:**
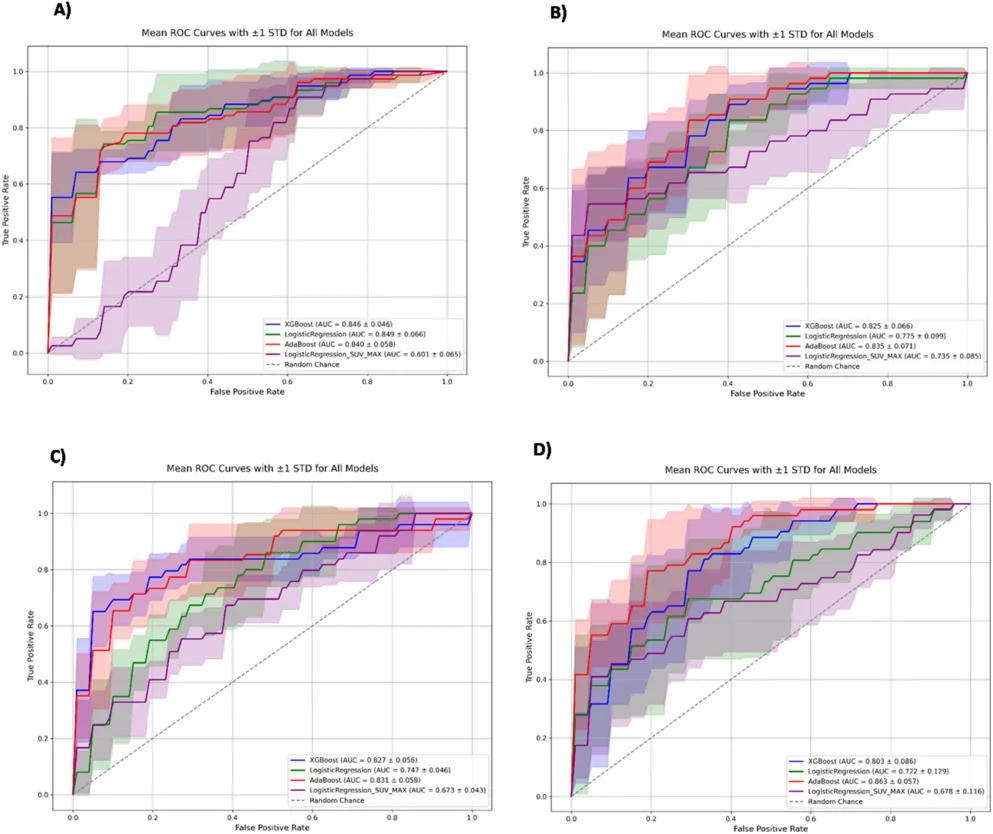
ROC curves and AUC values for different classifiers. Tree-based classifiers include AdaBoost (red) and XGBoost (blue). Logistic regression classifiers are shown in green, and the SUVmax-based logistic regression model is depicted in violet. (**A**) ROC curves for C-HL versus other subtypes (high-grade, NS-HL, DLBCL, and less common subtypes). (**B**) ROC curves for high-grade Non Hodgkinlymphoma versus other subtypes (C-HL, NS-HL, DLBCL, and less common subtypes) (**C**) ROC curves for nodular sclerosis versus other subtypes (C-HL, high-grade, DLBCL, and less common subtypes). (**D**) ROC curves for DLBCL versus other subtypes (C-HL, NS-HL, high-grade, and less common subtypes)

**Fig. 5:**
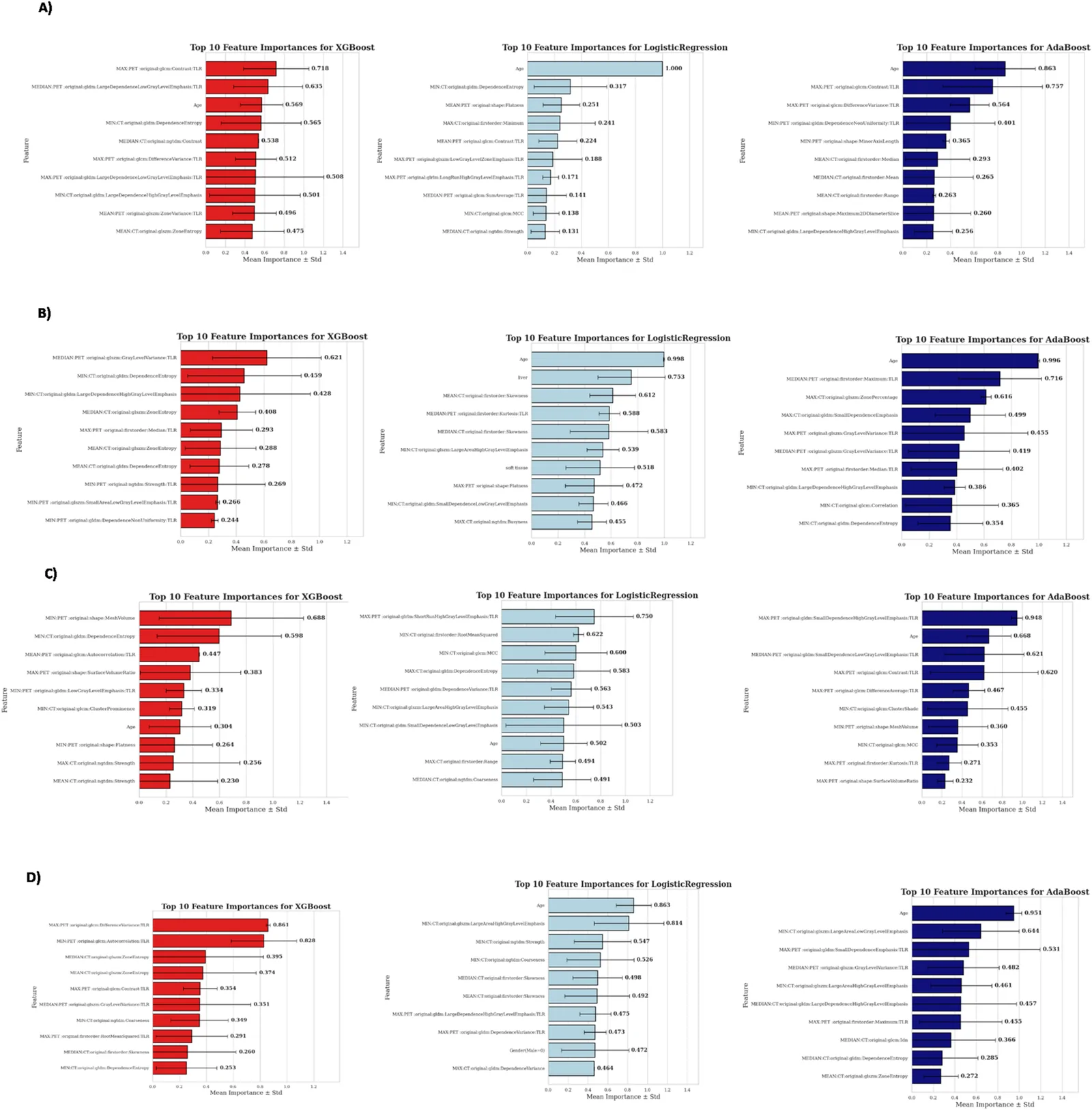
Top 10 most important features identified by each classification model in two classification tasks. Feature importances represent the mean values across cross-validation folds, and error bars indicate the standard deviation. Only features selected in at least two folds were included (**A**) C-HL vs. High-grade, NS-HL, DLBCL, and less common subtypes. (**B**) High-grade Non hodgkin lymphoma vs. C-HL, NS-HL, DLBCL, and less common subtypes. Each panel shows results for three models: XGBoost (left, red), Logistic Regression (middle, light blue), and AdaBoost (right, dark blue). (**C**) Nodular sclerosis vs. C-HL, High-grade, DLBCL, and less common subtypes (**D**) DLBCL vs. C-HL, NS-HL, High-grade, and less common subtypes

ROC curves (Fig. 4A) illustrate these findings, showing the clear superiority of multi-feature models over LR-SUV_MAX. Feature importance analysis (Fig. 5A) identifies the most discriminative radiomic features contributing to C-HL classification.

In distinguishing High-Grade NHL (Table 2), XGBoost achieved the highest accuracy (0.735 ± 0.075) and AUC (0.825 ± 0.066), surpassing Logistic Regression (accuracy 0.690 ± 0.097, AUC 0.775 ± 0.099) and AdaBoost (accuracy 0.723 ± 0.101, AUC 0.835 ± 0.071). Sensitivity values were moderate across models, with specificity consistently above 0.75. The LR-SUV_MAX model again showed the lowest performance metrics (accuracy 0.708 ± 0.029, AUC 0.700 ± 0.017). DeLong’s test results confirmed significant differences favoring XGBoost and AdaBoost over Logistic Regression and LR-SUV_MAX (*p* < 0.001) (Tables 2 and 3). ROC curves for this task (Fig. 4B) visually support these results. Feature importance rankings (Fig. 5B) highlight key features driving the models’ classification decisions.

**Table 3:** DeLong’s test results for AUC comparisons in lymphoma subtype classification

Subtype	Comparison	Z Statistic	*P*-value	Significant
C-HL	XGBoost vs. LogisticRegression	−1.460	0.1442	No
	XGBoost vs. AdaBoost	−0.322	0.7478	No
	XGBoost vs. LR-SUV_MAX	22.105	< 0.001	Yes
	LR vs. AdaBoost	1.228	0.2195	No
	LR vs. LR-SUV_MAX	23.201	< 0.001	Yes
	AdaBoost vs. LR-SUV_MAX	23.010	< 0.001	Yes
High-Grade NHL	XGBoost vs. LogisticRegression	5.113	< 0.001	Yes
	XGBoost vs. AdaBoost	−3.567	0.0004	Yes
	XGBoost vs. LR-SUV_MAX	8.643	< 0.001	Yes
	LR vs. AdaBoost	−7.340	< 0.001	Yes
	LR vs. LR-SUV_MAX	3.844	0.0001	Yes
	AdaBoost vs. LR-SUV_MAX	10.857	< 0.001	Yes
NS-HL	XGBoost vs. LogisticRegression	9.169	< 0.001	Yes
	XGBoost vs. AdaBoost	−1.407	0.1595	No
	XGBoost vs. LR-SUV_MAX	14.092	< 0.001	Yes
	LR vs. AdaBoost	−10.001	< 0.001	Yes
	LR vs. LR-SUV_MAX	5.008	< 0.001	Yes
	AdaBoost vs. LR-SUV_MAX	14.817	< 0.001	Yes
DLBCL	XGBoost vs. LogisticRegression	8.281	< 0.001	Yes
	XGBoost vs. AdaBoost	−5.539	< 0.001	Yes
	XGBoost vs. LR-SUV_MAX	10.749	< 0.001	Yes
	LR vs. AdaBoost	−12.300	< 0.001	Yes
	LR vs. LR-SUV_MAX	3.532	< 0.001	Yes
	AdaBoost vs. LR-SUV_MAX	14.392	< 0.001	Yes

Description: Results of DeLong’s test comparing area under the receiver operating characteristic curve (AUC) for XGBoost, Logistic Regression (LR), AdaBoost, and Logistic Regression with SUV features (LR-SUV_MAX) using final test data for Classical Hodgkin Lymphoma (C-HL), High-Grade Non-Hodgkin Lymphoma (High-Grade NHL), Nodular Sclerosis Hodgkin Lymphoma (NS-HL), and Diffuse Large B-Cell Lymphoma (DLBCL) versus other lymphomas. Z-statistics, p-values, and significance (*p* < 0.001) are reported

For NS-HL classification (Table 2), XGBoost demonstrated the best accuracy (0.832 ± 0.038) and AUC (0.827 ± 0.056), with AdaBoost performing comparably (accuracy 0.787 ± 0.033, AUC 0.831 ± 0.058). Logistic Regression showed decreased accuracy and AUC (0.677 ± 0.035 and 0.747 ± 0.046), and LR-SUV_MAX again had the lowest scores (accuracy 0.592 ± 0.051, AUC 0.638 ± 0.059). Sensitivity was highest for AdaBoost (0.753 ± 0.107), and specificity peaked for XGBoost (0.887 ± 0.098). Statistical testing showed significant superiority of XGBoost and AdaBoost over other models (*p* < 0.001) (Table 2). ROC curves (Fig. 4C) and feature importance patterns (Fig. 5C) further emphasize these distinctions.

In the classification of DLBCL (Tables 2 and 3), AdaBoost outperformed other models with an AUC of 0.863 ± 0.057 and accuracy of 0.742 ± 0.058, followed by XGBoost and Logistic Regression. The LR-SUV_MAX model had inferior performance (accuracy 0.708 ± 0.041, AUC 0.664 ± 0.065). Sensitivity and specificity were moderate across classifiers, with AdaBoost achieving the highest sensitivity (0.689 ± 0.151). DeLong’s test confirmed statistically significant performance differences favoring AdaBoost and XGBoost compared to LR and LR-SUV_MAX (*p* < 0.001) (Table 2). ROC curves (Fig. 4D) and feature importance (Fig. 5D) depict the discriminative capacity of these models.

**Fig. 6:**
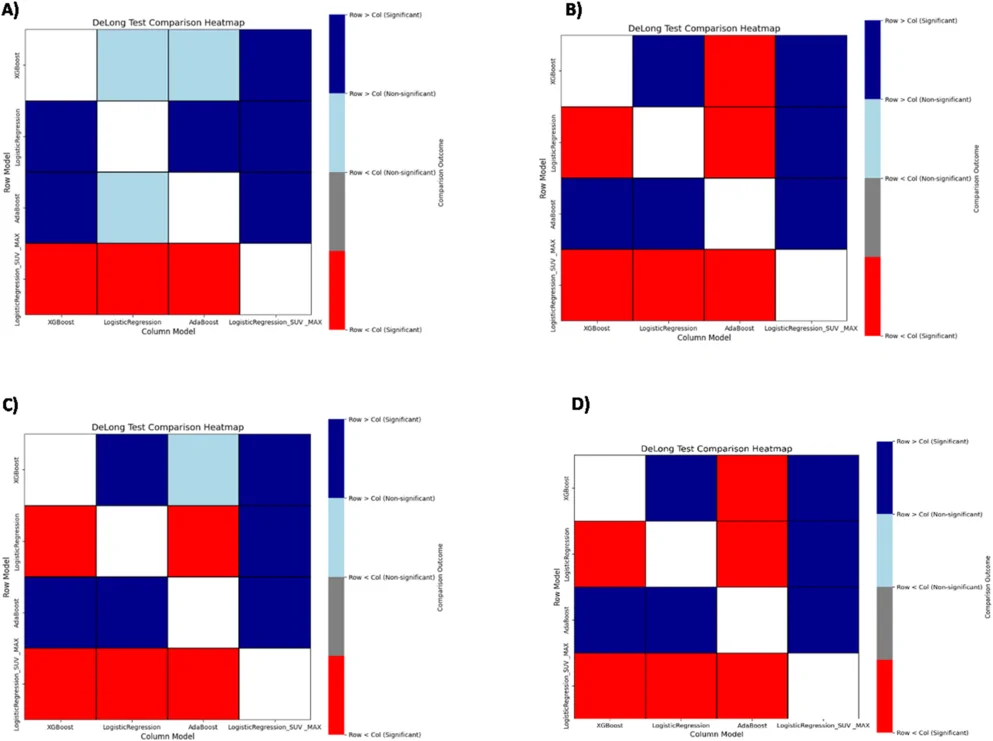
Heatmap illustrating pairwise AUC comparisons between classification models using DeLong’s test. Each cell shows whether the row model performed better, worse, or similarly to the column model, with color indicating the direction and statistical significance: dark blue (significantly better), light blue (better, not significant), gray (worse, not significant), and red (significantly worse). (**A**) for C-HL versus other subtypes (high-grade, NS-HL, DLBCL, and less common subtypes); (**B**) for high-grade Non Hodgkin lymphoma versus other subtypes (C-HL, NS-HL, DLBCL, and less common subtypes); (**C**) for nodular sclerosis versus other subtypes (C-HL, high-grade, DLBCL, and less common subtypes); (**D**) for DLBCL versus other subtypes (C-HL, NS-HL, high-grade, and less common subtypes)

**Fig. 7:**
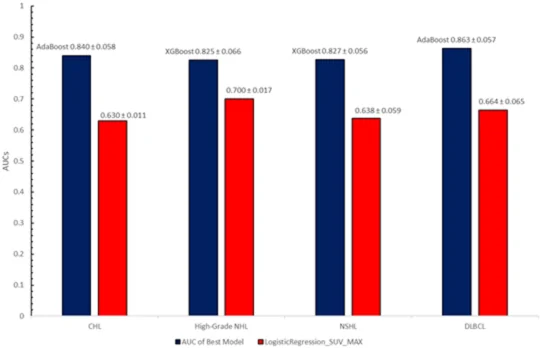
Comparison of AUC values for the best-performing model and the baseline LogisticRegression_SUV_MAX model across different lymphoma subtypes

The heatmap of pairwise AUC comparisons (Fig. 6) highlights that XGBoost and AdaBoost consistently outperform Logistic Regression and LR-SUV_MAX across lymphoma subtypes, with significance indicated by dark blue and red coloring. As shown in Fig. 7, LR-SUV_MAX consistently underperformed, highlighting the limitations of using SUV_max_ alone. The best-performing models achieved higher AUC values than this baseline across all classification tasks.

Feature importance analysis was performed for each classification task using three machine learning models: XGBoost, Logistic Regression, and AdaBoost. Across all tasks, Age emerged as a consistently important clinical variable. In addition, radiomic features derived from PET and CT images—particularly those related to GLCM, GLDM, and GLSZM texture matrices, as well as shape descriptors—were frequently selected across subtypes. In the case of C-HL, PET texture features (e.g., glcm: Contrast: TLR) and Age were most predictive. For High-Grade Non-Hodgkin Lymphoma, GLSZM features and liver involvement were prominent. In Nodular Sclerosis HL, shape and texture features from both PET and CT were relevant. Lastly, in DLBCL, PET-based GLCM features (e.g., Difference Variance: TLR), CT texture metrics, Age, and Gender were top contributors. Full rankings of the top 10 features for each model are shown in Fig. 5. The mean and standard deviation of the selected features’ importance values are presented in Fig. 5, with the complete list, including mean importance and standard deviations, provided in Supplementary Excel File 1.

### Survival Analysis

Ultimately, we obtained information on the 5-year survival of 74 patients, with 17 deceased, and for the 3-year survival, data was available for 110 patients, with 12 deceased. Table 4 illustrates the reported p-values for the significant radiomics and clinical characteristics that are associated with overall survival in both the 3- and 5-year intervals, based on the Mann-Whitney test. The complete list of significant features with their p-values for 3- and 5-year overall survival is available in Supplementary Excel file 2.

**Table 4:** Significant radiomics and clinical features associated with patient survival at 3 and 5 years, identified by Mann-Whitney test

Feature name	Category	Source	Stat. measure	*P*-value 5Y	*P*-value 3Y
Radiomics:					
Small area high gray level emphasis	GLSZM	SUV	Median	0.0181	0.0250
Gray level non uniformity	GLDM	SUV	Maximum	0.0131	0.00552
Gray level non uniformity	GLRLM	SUV	Maximum	0.0254	0.0116
Large area high gray level emphasis	GLSZM	SUV	Maximum	0.0237	0.0277
Sphericity	Shape	-	Minimum	0.0214	0.0366
10 Percentile	First order	SUV	Minimum	0.0398	0.0095
Maximum	First order	SUV	Minimum	0.0450	0.0028
Gray level non uniformity	GLSZM	SUV	Minimum	0.0187	0.0067
Small area emphasis	GLSZM	SUV	Minimum	0.0019	0.0142
Small area high gray level emphasis	GLSZM	SUV	Minimum	0.0174	0.0130
Strength	NGTDM	SUV	Minimum	0.0351	0.0080
Clinical:					
Stage	-	-	-	0.021	0.020
Age	-	-	-	0.0002	0.0060
Extra nodal involvement	-	-	-	0.0043	0.0017
Bone involvement	-	-	-	0.0077	0.0175
Spleen involvement	-	-	-	0.0156	0.0014

Abbreviations: GLCM: gray-level co-occurrence matrix, GLSZM: gray-level size zone matrix; SUV: standardized uptake value, Stat. measure: Statical measurement, P-value 5Y: P-value in 5-Y, P-value 3Y: Pvalue in 3-Y

## Discussion

This study aimed to evaluate the performance of machine learning models integrating ^18^F-FDG PET/CT radiomic features and clinical variables to help replace invasive biopsy in lymphoma patients. In the classification tasks, machine learning models utilizing multi-dimensional radiomic features consistently outperformed the SUV_max_-only Logistic Regression baseline model across all lymphoma subtypes. For instance, in classifying C-HL, Logistic Regression and AdaBoost models achieved accuracies of 77.5% ± 4.2% and 76.9% ± 4.8%, respectively, with AUC values close to 0.85. The SUV_max_-only model lagged behind substantially, showing an accuracy of 63.8% ± 2.5% and specificity as low as 49.3%. These results align well with previous studies. One study comparing PET radiomics and SUV_max_ for distinguishing PMBCL from C-HL reported a higher AUC for radiomics (0.87 vs. 0.78) [24]. Similarly, another study found that a Gradient Boosting model using radiomic features achieved better performance (AUC = 0.86, accuracy = 80%) than SUV_max_-based logistic regression (AUC = 0.79, accuracy = 70%) in differentiating FL from DLBCL [25]. These findings underscore the limited utility of SUV_max_ alone and the enhanced discrimination power of multi-feature radiomic models.

Similar patterns were observed in other subtypes, where XGBoost yielded the highest accuracy of 73.5% ± 7.5% and AUC of 0.825 ± 0.066 in High-Grade NHL classification, significantly surpassing Logistic Regression and the SUV_max_ model (*p* < 0.001, DeLong’s test). Similarly, For Nodular Sclerosis Hodgkin Lymphoma (NS-HL), XGBoost demonstrated superior performance with 83.2% ± 3.8% accuracy and an AUC of 0.827 ± 0.056, while AdaBoost achieved comparable results. In DLBCL classification, AdaBoost outperformed all other models, with an AUC of 0.863 ± 0.057 and accuracy of 74.2% ± 5.8%. These consistent findings are aligned with other research that highlighted the potential of 18 F-FDG PET/CT radiomics in lymphoma subtype classification as well as in differentiating lymphoma from other diseases such as sarcoidosis [19–23, 26]. Moreover, a study reported an accuracy of approximately 0.83 for both DLBCL and follicular lymphoma, with 0.94 accuracy for HL and 0.81 for mantle cell lymphoma using PET radiomics [27].

Across all models, patient age consistently stood out as one of the strongest predictors in every classification task. This was determined by averaging feature importance values across the five outer folds of our nested cross-validation (see Fig. 5). This observation aligns well with what clinicians already know about lymphoma epidemiology and prognosis.

Besides age, several radiomic features extracted from PET and CT images were repeatedly selected as important. Most of these were texture features derived from Gray Level Co-occurrence Matrix (GLCM), Gray Level Dependence Matrix (GLDM), and Gray Level Size Zone Matrix (GLSZM), along with various tumor shape descriptors.

For example, in the classification of classical Hodgkin lymphoma (C-HL) (Fig. 5A), PET texture features—particularly GLCM contrast normalized by TLR—together with patient age showed strong predictive power. This suggests that metabolic heterogeneity plays a crucial role in this subtype, a finding supported by previous studies reporting an AUC of 0.95 when using TLR radiomics to distinguish Hodgkin lymphoma from DLBCL [26]. Similarly, for High-Grade Non-Hodgkin lymphoma (Fig. 5C), GLSZM features combined with liver involvement emerged among the top predictors, likely reflecting the aggressive biology and extranodal spread typical of these tumors. In Nodular Sclerosis Hodgkin Lymphoma (NS-HL, Fig. 5C) and DLBCL (Fig. 5D), both tumor shape and texture features had significant predictive value. This highlights the importance of tumor morphology and intratumoral heterogeneity in differentiating lymphoma subtypes.

The Mann-Whitney test results presented in Table 4 indicate that SUV-based features were consistently associated with overall survival at both three- and five-year intervals. Among these features, two were first-order statistics. One was shape-related, and another was texture-based. Age emerged as one of the most significant clinical factors influencing patient survival. The median age of patients who died was 60 years for those who did not survive beyond five years. This is compared to 58 years for those who survived at least three years.

Notably, extranodal involvement—particularly in the spleen and bones—was a significant prognostic factor. Specifically, 12 out of 17 patients who did not survive more than five years had extranodal disease. Similarly, 9 out of 12 patients who did not survive beyond three years showed extranodal involvement. Previous studies have also highlighted the prognostic value of ^18^F-FDG PET/CT texture features in lymphoma patient survival [37–39].

The biological interpretation of these radiomic features further supports their clinical relevance. Texture features such as Gray Level Non-Uniformity (GLNU) from GLDM and GLSZM showed significant associations with overall survival at both 3- and 5-year intervals (*p* < 0.05). This indicates that higher heterogeneity in FDG uptake correlates with more aggressive disease phenotypes. Small Area High Gray Level Emphasis (SAHGLE), which captures focal regions of elevated metabolic activity, was predictive of poorer survival. This possibly reflects proliferative tumor hotspots or necrotic areas surrounded by metabolically active cells. Tumor shape, measured by sphericity, was significantly linked to survival outcomes. Lower sphericity values indicate irregular and invasive growth patterns that may predict worse prognosis. Additionally, first-order statistics such as the minimum value of the 10th percentile and maximum SUV enhanced the prognostic model. These findings suggest that extreme metabolic activity provides valuable information beyond average uptake measures.

Additionally, clinical features such as disease stage, patient age, extranodal involvement, and specific organ sites (bone and spleen) were statistically significant predictors of survival (p-values ranging from 0.0014 to 0.021). These findings align with current clinical understanding that advanced-stage disease and extranodal spread are associated with poorer outcomes. Integrating these clinical variables with robust radiomic biomarkers strengthens the model’s ability to stratify patient risk and supports more personalized treatment planning.

In summary, combining PET/CT radiomics with clinical data better classifies lymphoma subtypes than SUV_max_ alone. Key texture and shape features capture tumor heterogeneity and structure, making them valuable imaging biomarkers. These findings support the role of radiomics as a non-invasive adjunct for subtype stratification, particularly when biopsy is contraindicated or inconclusive.

Nonetheless, certain limitations must be acknowledged. Our study was conducted at a single center, which may limit generalizability. In addition, diagnostic variability among the multiple pathologists involved could have influenced the results. We decided to include rarer lymphoma subtypes to make sure our model covers the full range of cases doctors actually see. Although adding these less common types probably lowered the overall accuracy—because they have fewer examples and more variation—it’s important for building a tool that works well in real life. Moreover, the lack of subtype-specific survival analysis may have introduced bias, as survival outcomes vary considerably across lymphoma types.

The isotropic resampling to 2 × 2 × 2 mm³ for PET images (using trilinear interpolation) performed in this study may introduce minor interpolation-dependent texture artifacts. As noted in the IBSI guidelines, spatial resampling is necessary to achieve rotationally invariant texture features when the original voxels are anisotropic, since many texture algorithms assume equidistant neighboring voxels [40]. While we applied a consistent resampling strategy across the entire cohort following common PET radiomics practice, we did not perform dedicated robustness analysis to evaluate the sensitivity of features to different voxel sizes or interpolation methods. Future multi-center studies should include such sensitivity analyses to confirm feature stability.

Looking ahead, future studies should aim to validate these findings in larger and more diverse patient groups. Following radiomic features over time during treatment might help us better predict how patients will do and monitor therapy response. If these models continue to show promise, integrating them into clinical workflows through automated PET/CT analysis systems could support doctors in making quicker, more accurate decisions early in the diagnostic process.

## Conclusion

This study demonstrates that radiomic features derived from baseline ^18^F-FDG PET/CT scans, when normalized by tumor-to-liver ratios and analyzed using machine learning models, can significantly improve the classification of lymphoma subtypes compared to conventional SUV_max_-based metrics. Among the evaluated models, XGBoost and AdaBoost consistently outperformed logistic regression and the SUV_max_-based baseline across all subtype classification tasks, with statistically significant differences (*p* < 0.001). Furthermore, several radiomic and clinical features were found to be significantly associated with long-term survival, highlighting their potential role as non-invasive prognostic biomarkers. These findings support the integration of PET/CT radiomics into the diagnostic and prognostic workflows for lymphoma patients.

## Supplementary Information

Below is the link to the electronic supplementary material.

Supplementary File 1 (XLSX 10.9 KB)

Supplementary File 2 (XLSX 11.8 KB)

Supplementary File 3 (XLSX 11.8 KB)

Supplementary File 4 (XLSX 11.5 KB)

Supplementary File 5 (XLSX 12.3 KB)

Supplementary File 6 (XLSX 14.4 KB)

## Data Availability

The clinical studies used in this work are not available.
